# Building the graph of medicine from millions of clinical narratives

**DOI:** 10.1038/sdata.2014.32

**Published:** 2014-09-16

**Authors:** Samuel G. Finlayson, Paea LePendu, Nigam H. Shah

**Affiliations:** 1 Center for Biomedical Informatics Research, Stanford University, Stanford, California 94305, USA

## Abstract

Electronic health records (EHR) represent a rich and relatively untapped resource for characterizing the true nature of clinical practice and for quantifying the degree of inter-relatedness of medical entities such as drugs, diseases, procedures and devices. We provide a unique set of co-occurrence matrices, quantifying the pairwise mentions of 3 million terms mapped onto 1 million clinical concepts, calculated from the raw text of 20 million clinical notes spanning 19 years of data. Co-frequencies were computed by means of a parallelized annotation, hashing, and counting pipeline that was applied over clinical notes from Stanford Hospitals and Clinics. The co-occurrence matrix quantifies the relatedness among medical concepts which can serve as the basis for many statistical tests, and can be used to directly compute Bayesian conditional probabilities, association rules, as well as a range of test statistics such as relative risks and odds ratios. This dataset can be leveraged to quantitatively assess comorbidity, drug-drug, and drug-disease patterns for a range of clinical, epidemiological, and financial applications.

## Background & Summary

**Figure 1:**
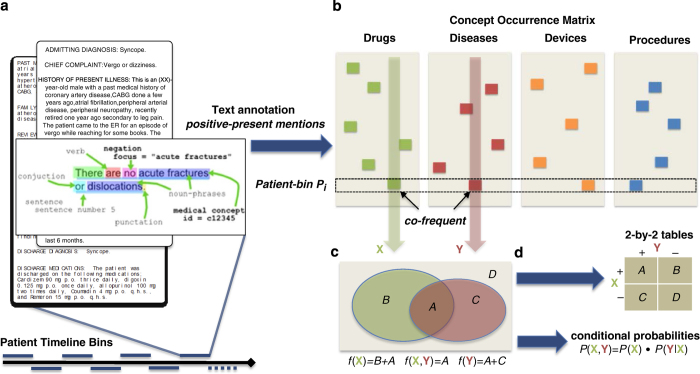
Workflow Architecture. The architecture of our workflow starts with (**a**) patient notes that are grouped together based on their nearness in time. Given the patient timeline bins, clinical terms are recognized from the notes and recorded into (**b**) the clinical concept occurrence matrix, which is scanned for (**c**) counting pairwise the frequency and co-frequency of concepts. This data can be used to calculate (**d**) contingency tables and Bayesian probability estimates. For example, the concept X has a frequency of *f*(X) and is pairwise co-frequent with concept Y exactly *f*(X,Y) times.

The widespread adoption of Electronic Health Records (EHRs) has enabled the longitudinal collection of patient health information at an unprecedented granularity and scale. As outlined by Jensen *et al.*^1^, efforts to mine this data show promise to impact nearly every aspect of healthcare. Nevertheless, these efforts are hindered, in part due to ethical and legal restrictions to data access. By tabulating clinical concept co-occurrences across millions of patient records and over a range of time windows (Figure 1), we provide rare quantitative glimpse into the degree of inter-relatedness of medical entities in large-scale clinical data.

Knowledge of such inter-relatedness can be readily leveraged for improving fundamental methods underlying a wide range of use cases, such as patient clustering^2^ and outcome prediction^3^, analysing comorbidity patterns^4,5^, and cohort querying for clinical trial design^6^. We provide here a brief overview of several areas that could employ the degree of inter-relatedness data we present.

Clinical concept co-occurrences can be used by clinical decision support (CDS) systems. The INTERNIST-1 system, its successor the Quick Medical Reference (QMR), and other diagnostic CDS utilities such as MYCIN and Pathfinder have been developed to aid physicians in diagnosing patients given their collections of symptoms^7–11,7–11,7–11,7–11,7–11^. Such systems generally depend on the painstaking manual development of knowledge bases to associate symptoms with diagnoses with varying degrees of confidence^12^. The website for one new commercial CDS system, for example, advertises the application of more than 20,000 physician hours in the construction of such a graph^13^. Beyond the human burden for the initial population of such knowledge bases, manually curated datasets suffer from the rapid turnover in expert knowledge and the state of the literature, which leads to challenges in their upkeep or results in rapid out-dating^9,14^. By leveraging co-occurrence data from real patient records, CDS systems could be populated with necessary conditional probabilities in a manner that is fast, scalable, easily updated, and by nature reflective of the patterns truly observed in clinical practice.

Clinical data allow probing population-wide comorbidity patterns over thousands of variables. As described by Valderas *et al.*^15^, although the notion of comorbidity is difficult to precisely define, the ability to quantitatively assess disease concurrence is of major interest within clinical, epidemiological, and financial health service contexts. Commonly, an individual’s comorbidities are assessed by means of patient stratification or cumulative disease burden index, such as the Charlson Index^16,17^. Though specialized versions of such indices have been developed, such as transplantation^18,19^, the process of using custom studies to derive disease-specific comorbidity indices is un-scalable. In contrast, data-driven approaches applied to comprehensive EHR datasets, such as that presented here, can learn comorbidity patterns across thousands of conditions^4,5,20,21^, thus complementing existing efforts.

Co-occurrence based analyses, which includes unstructured data sources in particular, can also enable new insights. For example, co-occurrence based disproportionality analysis across drug-disease pairings is employed for adverse drug event (ADE) detection in the field of computational pharmacovigilance. While many drug surveillance efforts focus on mining spontaneous reporting databases, given the underreporting in such databases^22^ and concern that coded data may miss >90% of adverse drug events^23^, recent efforts utilize unstructured EHR data for active pharmacovigilance^24–26,24–26,24–26^.

Examining disease co-occurrence patterns can enable the computational discovery of novel human health phenotypes^4^. Recent work has shown that many diseases classified historically as single entities are actually collections of many distinct phenotypes that may not match traditional disease boundaries^20,27^. Unsupervised learning methods applied to population-wide, comprehensive clinical datasets can redefine subtyping of existing diseases, and discover unrecognized phenotypes^28–30,28–30,28–30^. Our data can serve as the basis for building drug-drug, drug-disease, and disease-disease networks that can be utilized in such analyses.

The advent of larger datasets, including claims data, has given rise to high dimension propensity score methods that incorporate large numbers of variables for improved performance^31^. As the feature space for such analyses grows, so does the risk of latent instrumental or collider variables that could introduce Z- or M-biases^32^. Our co-occurrence counts can be used to infer latent dependencies between millions of clinical variables, which could spur the development of improved feature selection for high dimensional propensity scoring. Finally, inferred dependencies from our data could be used to assess the data assumptions of commonly used statistical methods, such as regression analysis, which commonly assumes feature independence.

## Methods

### Data source

We used clinical notes from the Stanford Translational Research Integrated Database Environment (STRIDE)^33^. The STRIDE dataset contains 20 million unstructured clinical notes from 1.2 million patients, collected over a 19-year span. The notes comprise a combination of pathology, radiology, and transcription reports. We limited our calculation to notes from patients whose records contained a minimum of 10 notes spanning a time window of at least one year. The result was a raw set of 14 million text notes from 260 thousand patients.

### Note binning

Each note contains terms that may be counted as co-occurring. However, we also employ a broader definition of concurrency by splicing together the contents of ‘bins’ of notes that are found to appear within a certain timeframe in a given patient’s record. Figure 1a depicts how a single patients’ set of notes can be spliced together over such bins.

Each note from STRIDE is time-stamped. For each patient, we used the time-stamps to hash notes into their temporal bins. Binning was accomplished via a simple hashing formula: For a patient *p* and bin width *w*, we note *t_0_* (time of first record) and generated a random real number 0≤*r*≤*w*. We then defined a patient-specific hashing function operating on *w, r,* and a note’s time *t* (the note’s delta from *t*_*0*_):

$$BIN_{p}(w,r,t)=\left\lfloor \frac{t+r}{w}\right\rfloor$$

For example, if *w*=7, then a note having a timestamp more than seven days later would necessarily be assigned a larger bin number. The random offset, *r*, was added to avoid potential global timestamp recording biases (e.g., a gone for the weekend effect).

For each patient’s longitudinal health record, we consider 1, 7, 30, 90, 180, 365, and ∞-day bins. The bins are used to aggregate the frequency counts in two ways: (1) per bin, or (2) per patient. In the per-bin scenario, each relevant bin contributes a count; thus, one patient can contribute many counts. In the per-patient scenario, each patient contributes at most one count, no matter how many of his or her bins relate. Note that in the ∞-day bin these two scenarios are identical because each bin represents an entire patient record. Thus, each row in Figure 1b either represents a bin or a patient depending on the aggregation scenario.

### Term extraction

To extract the clinical concepts contained within each unstructured clinical note, we utilized the approach described previously by LePendu *et al.*^24^ See Figure 1a for an illustration of the concept recognition method. We first find mentions of terms from a dictionary compiled from 22 clinically relevant ontologies, such as SNOMED-CT and MedDRA. We apply a series of syntactic and semantic suppression rules to create a clean lexicon from these ontologies. We keep terms that are predominantly noun phrases based on an analysis of MEDLINE abstracts; we remove uninformative phrases based on term frequency analysis of over 50 million clinical documents from the Mayo clinic; and we suppress terms having fewer than four characters because these tend to be ambiguous abbreviations^34–37,34–37,34–37,34–37^. Finally, NegEx regular expressions are used to flag negative mentions (e.g., ‘myocardial infarction was ruled out’) and to determine if a term is mentioned in the history or family history section of the note^38,39^. We map the terms to concepts using the same 22 ontologies. During this term-concept mapping we identify ambiguous terms that belong to more than one semantic group (drug, disease, device, procedure) and suppress their least likely interpretation. For example ‘clip’ is more likely to be a device than a drug in clinical text, so we suppress the interpretation as ‘Corticotropin-Like Intermediate Lobe Peptide. Further details on the text processing are found in LePendu *et al.*^24^

We filtered the list of terms by further applying a list of common stop words, as detailed in the Data Records section below. We additionally removed those terms that were shorter than four characters in length, as well as a manually curated list of terms that are homonymous for first or surnames. This final list of terms from each note was then indexed using Lucene^40^, and combined with the other notes in the same bin.

### Estimating the accuracy of the text processing pipeline

We determine the accuracy of the event identification using a gold standard corpus from the 2008 i2b2 Obesity Challenge. This corpus has been manually annotated by two annotators for 16 conditions and was designed to evaluate the ability of NLP systems to identify a condition present for a patient given a textual note. We extended this corpus by manually annotating 9 additional outcomes. The list of 25 conditions evaluated is listed in supplemental information of LePendu *et al.*^24^

Using the set of terms corresponding to the definition of the concept of interest and the set of terms recognized by our annotation workflow in the i2b2 notes, we evaluate the sensitivity and specificity of identifying each of the conditions. Overall, our condition identification has 74% sensitivity and 96% specificity. Further details are found in LePendu *et al.*^24^

### Co-frequency counting

A parallel processing approach was used to compute the co-frequencies on the terms within each bin (Figure 1b,c). This step was the most computationally expensive in the pipeline, being a *O*(n*m^2^) computation, where *n* is the total number of bins and *m* is the number of terms found within each bin. For example, for *w*=7, *n*=5.5 M and *m*=200, so the computation examines on the order of 220 billion pairs. A single pass over that many pairs takes approximately 9.5 h on a 20-core machine with 117GB of RAM. Table 1 lists summary statistics for each bin width.

Additionally, we provide a list of co-frequency counts computed over clinical concepts. For concept-level counting, we limited our data to terms that could be unambiguously mapped onto drugs or diseases. We then normalized all drugs to their ingredients using RxNorm, and normalized remaining terms to clinical concepts. We then counted the co-occurrences of the resulting concepts in the same manner as we counted the co-frequent terms. Finally, we removed rows from all files that contained counts of size 100 or fewer.

## Data Records

The clinical text frequency dataset represents term and concept frequency counts over notes from 261,397 patients, computed over a range of bin widths (Data Citation 1). Summary data for the number and average size of the bins in each record are found in Table 1. Files are stored in tab-delimited format as outline below. To minimize file size, terms and concepts are stored using unique term and concept IDs; the keys are found in Data Record 3. We also provide Python scripts to assist in decoding these files (see Usage Notes). Filenames and specifications are found in the README.txt that accompanies the dataset. We uploaded the data to the Dryad Digital Repository (datadryad.org). Please see Data Citations for details.

### Data record 1—co-frequency counts

The co-frequency counts are stored in 28 text files, each consisting of three tab-delimited columns. A row in each file represents a unique term-term or concept-concept pair and its counts, aggregated on a per-bin or per-patient basis with a given bin width *w*. Terms and concepts are encoded using unique ID numbers, the maps for which are found in Data record 3.

### Data record 2—singleton frequency counts

Singleton frequency counts are stored in 16 text files, each consisting of two tab-delimited columns. A row in each file represents a unique term or concept and its counts, aggregated on a per-bin or per-patient basis. Note that per-patient singleton counts are independent of bin width, and as such are only represented by a single file. The per-bin singleton counts, in contrast, are impacted by bin width and are as such represented by seven files, one per value of *w*. As with the co-frequency counts, terms and concepts are represented by integer term and concept IDs.

### Data record 3—term and concept ID mappings

**Figure 2:**
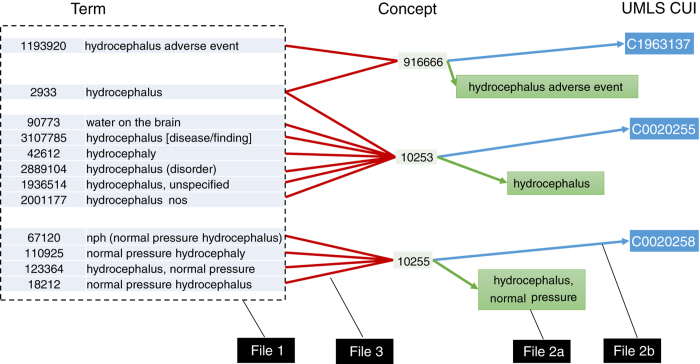
Mappings among terms and concepts. The figure explains the mappings that can be used to decode the frequency files stored in records 1 and 2. We use a subset of terms related to ‘hydrocephalus’ to demonstrate the mapping of terms (File 1) to concepts and UMLS CUIs. Terms map onto concepts in a many-to-many fashion (File 3). Concepts map onto CUIs in a one-to-one fashion (File 2b) and have an associated string for human readability (File 2a).

This record contains mappings that can be used to decode the frequency files stored in records 1 and 2. The relationships captured in the different files are further explained in Figure 2. We also provide in this record the list of stop word strings that were repressed from the analysis.

#### File 1—term ID definitions

Each row pairs an integer term ID number with the string that it encodes in our dataset. For our purposes, a term is defined as a unique string.

#### File 2a—concept ID definitions: concept ID to string dictionary

Each row represents an integer concept ID and actual concept it represents in string form and the Unified Medical Language System (UMLS) Concept Unique Identifier (CUI) for that concept.

#### File 2b—concept ID definitions: concept ID to CUI dictionary

Each row represents an integer concept ID and the UMLS CUI for that concept.

#### File 3—term to concept dictionary

A many-to-many mapping of term IDs to concept IDs. Any terms whose mapping onto concept is not listed was suppressed from the concept co-frequency counts.

#### File 4—stop words

The list of words treated as stop words in the analysis. The stop words list also contains the list of terms that are homonymous for first or surnames, which are excluded from the counting.

## Technical Validation

The annotation pipeline has been previously validated, as described in LePendu *et al.*^24^ We used simulation to assess the accuracy of our co-frequency counting methodology. We wrote a program that generates known term bins along with their correct co-occurrence counts. We simulated over 1,000 random ‘notes’ for 100 randomly simulated patients and computed the bins, and calculated the co-frequency counts at the bin and patient level using two different methods. We first wrote a single-threaded python script to take the simulated notes and count the co-frequencies basically duplicating the functionality of the system, but without using any parallelization. In parallel we ran co-frequency counter using Spark that used a different approach to parallelization than the one we used in the manuscript. The co-frequency counts from the two methods agreed perfectly with the parallelized method we implemented. Because we are calculating co-frequencies at a large scale using a distributed process, we focused on validating the transformation of the annotated text into bins, and into co-frequencies using a simulated dataset (where the answer is known) and two different approaches for comparison.

## Usage Notes

### Mapping terms to concepts and strings to CUIS

For convenience, we provide Python scripts, ‘decode_cofreqs.py’ and ‘decode_singlets.py’ that can be used to transform the term and concept IDs from a co- or singleton-frequency file into their corresponding strings and CUIs. These scripts can be adjusted or incorporated into an analysis pipeline, and follow the form:

load lookup table into map M, s.t. M[ID] → string for line in frequency file: extract term IDs x, y and count c print M[x] \t M[y] \t c

These scripts require only a Data Record 1 frequency file and a Data Record 2 dictionary file to complete the mapping. Example command line usage for the scripts is found within their header comments.

### Computing contingency tables

**Figure 3:**
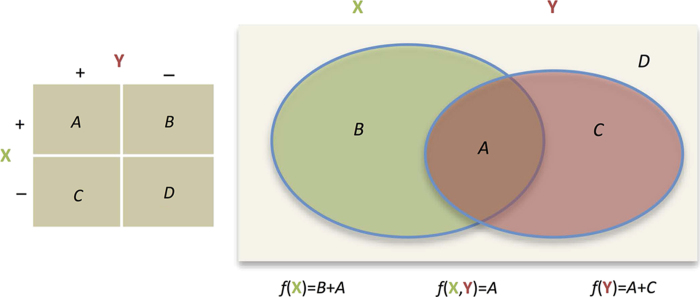
Filling out the 2-by-2 contingency table.

Co-occurrence data can be leveraged to build contingency tables (Figures 1c,d and 3). Contingency tables can be directly utilized in statistical tests, such as odds ratio, relative risk, the G-test, enrichment analysis and the chi-squared test^41^. In constructing contingency tables, our co-frequency counts can be utilized for hypothesis testing across many variables that are typically missing from large observational datasets, which can in turn motivate traditional research studies. Obviously multiple hypothesis testing becomes an issue that needs to be addressed^42^.

For example, in Figure 3, for two terms or concepts X and Y, their co-frequency is denoted *f*(X,Y) and would go into cell ‘*A*’ of the 2-by-2 contingency table. Visualized as a Venn diagram, cell ‘*B*’ is the frequency of X minus the intersection: *f*(X)−*f*(X,Y). Cell ‘*D*’ of the contingency table is calculated by subtracting *A*, *B*, and *C* from the universe *N*, which is the number of bins for the given width (row 1 of Table 1). Specifically, D=*N*−(*A*+*B*+*C*) or D=*N*−[*f*(X)+*f*(Y)]+*f*(X,Y). If computing over per-patient counts, use the ∞-day bin for *N*, which is the number of patients (*N*=261,397). The resulting table can then be plugged into any standard statistical package.

### Estimating bayesian probabilities

The data we present can also be leveraged in Bayesian networks^43^ (Figure 1d), which have been described in the literature as integral components of clinical decision support systems^7,8,44–46,44–46,44–46^. The basic Bayesian probabilities that form the basis for such methods can be inferred directly from our data. For example, given two clinical terms X, Y and a total number *N* of bins or patients (see section on contingency tables), one might compute:

$$P\left(X\right)=\frac{f(X)}{N}P\left(X,Y\right)=\frac{f(X,Y)}{N}P\left(Y|X\right)=\frac{P(X,Y)}{P(X)}$$

These probabilities can then be used to make additional estimates and inferences. For example, the lift of the association rule X→Y, which functions as a measure of statistical dependence between X and Y, can be computed as follows:

$$Lift\left(X\to Y\right)=\frac{P(Y|X)}{P(Y)}$$

The lift could then be utilized, for example, as a filter to suppress spurious findings in a disproportionality analysis or to assist in identifying M- or Z- bias in propensity score or regression models^31,32^. Our co-frequency counts can be used to construct networks of concept-concept associations in healthcare data, which can be employed to detect and eliminate variables that may introduce error in healthcare propensity models.

### Defining groups of related features

Statistical models to learn clinical phenotypes must incorporate a large number of variables, many of which are highly collinear^32^. Similar to using lift, one might leverage co-frequency counts as a component of a group LASSO penalty^47^ in building better phenotyping models. Regularized regression methods such as the LASSO can be employed to reduce the feature space and enhancements such as the group LASSO can enforce sparsity both within and between defined sets of variables. Co-frequency counts can be used to define the groups of highly correlated variables over which the group LASSO penalty is applied. Similar approaches can be employed in the construction of statistical models with applications other than phenotyping, such as outcome prediction or risk assessment—in fact, any model that is trained over large numbers of clinical variables and is subject to assumptions of feature independence may benefit from a quantitative characterization of feature co-linearity from our data.

### Improved enrichment analysis

Enrichment Analysis, which determines whether the Gene Ontology (GO) terms about specific biological processes, molecular functions, or cellular components are over- or under-represented in the annotations of the genes considered differentially expressed^48^, is commonly used to gain insight into the biological significance of alterations in gene expression levels^49^, as well as to profile other genome scale data such as tissue-microarrays^50,51^.

Recently, enrichment analysis has been performed using disease ontologies, such as SNOMED-CT, as well as being applied to other datasets of interest, such as patient cohorts. For example, by annotating protein mutations with disease terms, Mort *et al.*^52^ identified a class of diseases—blood coagulation disorders—that are associated with a significant depletion in substitutions at O-linked glycosylation sites. In another example, enrichment analysis was used detect specific co-morbidities that have an increased incidence in *rheumatoid arthriti*s patients, a topic of recent discussion in the literature and considered essential to provide high quality care^53–55,53–55,53–55^.

Among the different limitations of enrichment analysis, a crucial one is the handling of dependence between annotations, e.g., annotation of an EHR with *hypertension* and *lisinopril* or *diabetes* are not independent because lisinopril is a drug that treats hypertension and diabetes is a common co-morbidity. The dependencies among annotations with terms from different branches of the GO are well known^49,56,57^. For example, genes annotated with the molecular function of *electron transport* are also annotated with the biological process of *cellular respiration* because *electron transport* occurs during *cellular respiration*. However, the dependencies between occurrences of medical concepts in patient records are not well quantified.

Methods to account for dependency among terms typically rely on co-occurrence frequency of terms in an annotation corpus. The intuition is that we can increase the ‘expectedness’ of a term based on the co-occurrence of that term with other terms in a large reference annotation set. Using the co-occurrence of the two terms, we can quantify the increased expectation and adjust the *P*-values accordingly. The dataset we provide allows researchers to account for annotation dependency via adjusting the enrichment calculation, by accounting for the overlap of two sets X (items annotated with *x*) and Y (items annotated with *y*) if the intersection A (items annotated with *x and y*) is known, as described by Grossman *et al.*^58^

In summary, electronic health records represent a rich and untapped resource in analysing the true nature of clinical practice. EHR-powered research is limited by privacy and security concerns that limit data sharing. While these limitations make sharing of our raw data impossible, we provide co-occurrence counts derived from processed clinical notes. These co-occurrence matrices can be mined directly for co-occurrence patterns of interest, can be leveraged to improve the performance and generalizability of statistical models with a large number of features, and can enable improved enrichment analyses of patient cohorts.

## Additional information

**How to cite this article:** Finlayson, S. G. *et al.* Building the graph of medicine from millions of clinical narratives. *Sci. Data* 1:140032 doi: 10.1038/sdata.2014.32 (2014).

## Supplementary Material



## Figures and Tables

**Table 1:** Summary statistics for each bin width.

	**1-day**	**7-day**	**30-day**	**90-day**	**180-day**	**365-day**	**∞-day**
**No. Bins**	7,334,261	5,571,972	3,969,069	2,716,892	2,014,460	1,417,462	261,397
**No. Notes/Bin**	1.99	2.62	3.67	5.36	7.24	10.28	55.76
**No. Terms/Bin**	169.48	200.70	246.12	305.91	363.36	447.20	1,417.36
**No. Concepts/Bin**	41.60	48.84	59.12	72.42	85.28	104.38	332.37
As bin width increases towards infinity, the total number of bins decreases, while the number of terms and concepts contained within each bin increases. The number of bins in the ∞-day window (261,397) is equal to the total number of patients.							
